# Weak population structure and no genetic erosion in *Pilosocereus aureispinus*: A microendemic and threatened cactus species from eastern Brazil

**DOI:** 10.1371/journal.pone.0195475

**Published:** 2018-04-09

**Authors:** Gulzar Khan, Paulianny M. Ribeiro, Isabel A. S. Bonatelli, Manolo F. Perez, Fernando F. Franco, Evandro M. Moraes

**Affiliations:** Departamento de Biologia, Universidade Federal de São Carlos, Sorocaba, São Paulo, Brazil; Chinese Academy of Sciences, CHINA

## Abstract

Succulent cacti (Cactaceae) are among the most threatened taxonomic groups assessed to date. Here we evaluated the genetic diversity and population structure of a narrow endemic columnar cactus *Pilosocereus aureispinus*. This species is only found in a small area of c. 300 km^2^ of rocky savanna from eastern Brazil and it is currently classified as vulnerable (VU) on the International Union for Conservation of Nature (IUCN) red list. Eight microsatellite loci were genotyped for 91 individuals from four localities of the known *P*. *aureispinus* range. In contrast with expectations for narrow endemic species, we found relatively high levels of genetic diversity (e.g., *H*_E_ = 0.390 to 0.525; *H*_O_ = 0.394 to 0.572) and very low population structure based on the variation of six loci. All the analyzed individuals were clustered in one unique genetic group in assignment tests. We also generated the sequences of two plastid markers (*trnT-trnL* and *psbD-trnT*) and found no variation on a subsample of 39 individuals. We used Landsat 8 images and Normalized Difference Vegetation Index to estimate a potential extent of occurrence of c. 750 km^2^ for this species. Our results showed that *P*. *aureispinus* is not suffering from erosion of nuclear genetic variability due to its narrow distribution. However, we advocate that because of the extremely limited extent of occurrence, the ongoing anthropogenic disturbances in its habitat, and phylogenetic distinctiveness of *P*. *aureispinus*, this species should be classified as endangered (EN) on the IUCN Red List.

## Introduction

Cactaceae is among the most threatened taxonomic groups assessed to date. Approximately 31% of the total 1,478 evaluated species is threatened with extinction [1]. The major factors of extinction risk in Cactaceae are the collection of fresh plants and seeds for horticultural trade, mining operations, livestock ranching and smallholder annual agriculture [1]. The Cactaceae are rich in rare and narrow endemic species, which are expected to be more sensitive to extinction events due to reduced population sizes and spatial isolation [2, 3]. Such populations are expected to experience genetic erosion, i.e. to have reduced genetic diversity compared to more widely distributed species [4,2,5,6], resulted from high genetic drift and inbreeding within populations and reduced gene flow between them [2,7]. Consequently, the reduced heterozygosity leads to a reduction in individual fitness and undermines population viability in a relatively short evolutionary time. Additionally, a decline in allelic richness may also limit the ability of populations to overcome new selective pressures [2, 3]. To promote greater objectivity and transparency to assess the conservation status of a species, the International Union for Conservation of Nature (IUCN) has developed a guide [8] in which the amplitude of the geographic distribution and population size are assumed to have an important impact on extinction risk. The consensus is that the restricted distribution is a sufficient condition to classify a taxon as threatened species [8]. Eastern Brazil is one of the most important centres of cacti diversity [1], with about 40 genera and 227 species [9], including habitats in Caatinga, Brazilian Atlantic Forest, and Cerrado biomes. This region comprises a high endemism rate, with about 77% of the Cactaceae species being endemic [10]. *Pilosocereus* (Subfamily Cactoideae, Tribe Cereeae) is one of the most diverse cactus genera of Eastern Brazil (44 species subdivided into two subgenera). This genus includes many species patchily distributed along mountain ranges or highlands within the Cerrado biome in South America [11]. Most of the species have fragmented distribution, restricted to the *campo rupestre* landscapes, a grassland mosaic and associated vegetation on the rocky outcrops of eastern and central Brazil embedded into a forest matrix [12]. Among these species, *P*. *aureispinus* is currently known from only five sites of arenitic rock outcrops within an area of c. 300 Km^2^ [13] on the eastern shore of the São Francisco River, in municipalities of Ibotirama and Oliveira dos Brejinhos, Bahia state of Brazil [11]. Although the Cerrado vegetation predominates in this area, it is located in a semi-arid region bordering the Caatinga biome, where *Pilosocereus* has a high importance in terms of taxonomic diversity. This species is included in the IUCN Red List of Vulnerable Species [13] due to its restricted distribution, threats of habitat loss due to its proximity to highways, and frequent fire on the landscape. Another factor that demands conservation attention to *P*. *aureispinus* is its phylogenetic distinctiveness. Although *P*. *aureispinus* has been classified as a member of the Aurisetus species group (one of the five informal taxonomic groups of the subgenus *Pilosocereus* [11, 14]), it has been recovered in recent molecular phylogenies as an early divergent taxon, sister of the subgenus *Pilosocereus* [15, 16].

*Pilosocereus aureispinus* has a shrubby habit with erect cladodes branched at ground level, up to two meters in height. The epidermis is dark green with translucent spines at the base, ranging from gold to ferruginous, with the presence of long white trichomes in its areolas. The flowers are robust with colors ranging from pink to white and nocturnal anthesis. Considering these floral characteristics their possible pollinators are bats, as observed in other *Pilosocereus* species with similar floral morphology [17]. The seeds are elongated-shape with conical testa-cells, a feature possibly related to dispersion by ants [11]; bats and birds are also thought to be effective seed dispersal vectors [10].

In the present study we investigated the genetic diversity and population genetic structure of *P*. *aureispinus* in order to inform conservation actions for *P*. *aureispinus*. To access genetic diversity, we genotyped eight nuclear microsatellite loci in 91 individuals from four localities and sequenced two cpDNA markers (*tnrT-trnL* and *psbD-trnT*). We investigate the potential extent of occurrence of *P*. *aureispinus* using satellite images (Landsat 8) and Normalized Difference Vegetation Index. As *P*. *aurespinus* occurs in a small region of fragmented landscape, our expectations were to find low levels of genetic diversity along with some degree of population structure. Further, as plastid DNA is more influenced by genetic drift due to its smaller effective population size, and likely experiences less gene flow than nuclear DNA [11], we expect to find decreased variation and higher population structure in plastid DNA than in the microsatellite dataset.

## Materials and methods

### Ethics statement

The known populations of *Pilosocereus aureispinus* only occur in unprotected areas, and all samples utilized here were collected on private lands with permission of the landowners and with legal authorization to EMM of the Instituto Chico Mendes de Conservação da Biodiversidade (ICMBio) in accordance with the Brazilian law (Permit Number: Sisbio # 2022310).

### Plant sampling and DNA extraction

**Fig 1:**
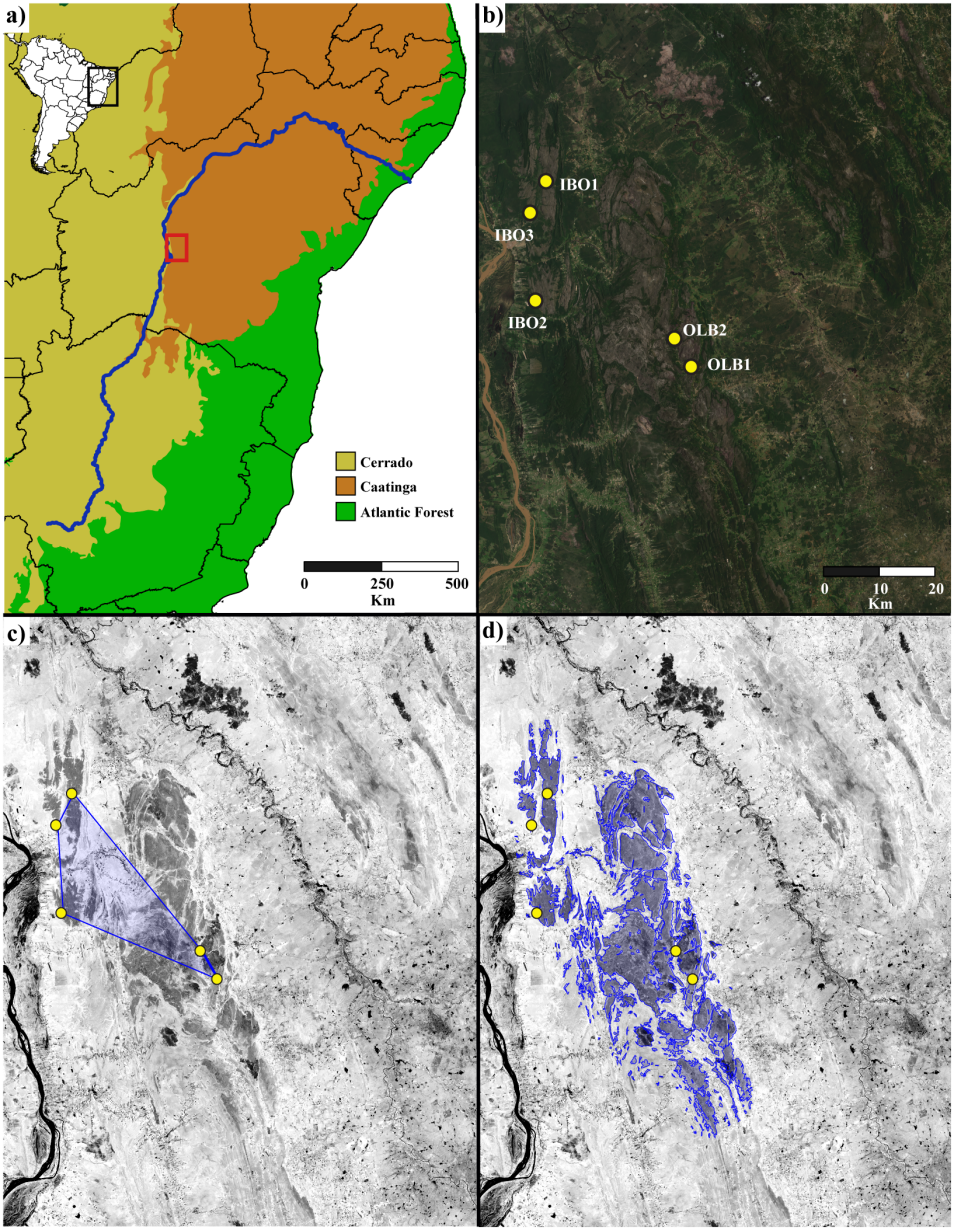
Geographic distribution of known occurrence sites for *P*. *aureispinus* (sampled sites are shown coded according to Table 1) and extent of occurrence estimates. a) Map of eastern Brazil showing the studied area (red inset), the São Francisco River course in blue and main biomes. b) Natural colour Landsat 8 image. c) NDVI image with healthy vegetation in light grey and exposed soil in dark grey showing the minimum convex polygon encompassing all occurrence points in blue. d) NDVI image showing, inside the blue contour, the estimated extent of occurrence by setting a NDVI threshold. Locality and geographic coordinates of the samples IBO1 and IBO2: Ibotirama, Bahia state, S12°05’ W43°09’ and S12°16’ W43°10’, respectively; OLB1 and OLB2: Oliveira dos Brejinhos, Bahia state, S12°22’ W42°55’ and S12°20’ W42°56’, respectively. The unsampled site IBO3 is located at Ibotirama, Bahia state, S12°13’ W43°18’.

We sampled 91 individuals from four of the five known occurrence sites of *P*. *aureispinus* (Fig 1). Distances among sampled sites ranged from 148 km (IBO1 and OLB1) to approximately 5 km (OLB1 and OLB2). In order to minimize the probability of sampling clones, a distance of at least ten meters were maintained among sampled individuals. Samples were deposited in a zip-bag containing silica gel in the field and frozen upon arrival in the laboratory. Specimens were identified according to [11, 14]. Genomic DNA was extracted from root tissues by using commercial Dneasy Plant Mini Kit (Qiagen) and quantified through electrophoresis on 1% agarose gel.

### Molecular techniques

Two plastid markers (*trnT-trnL* and *psbD-trnT*) were sequenced based on previous screening variation studies in *Pilosocereus* [18] in a subsample of 39 individuals. Amplification of the *trnT-trnL* intergenic spacer regions followed the PCR conditions and primers described in [18], while for *psbD-trnT* regions we used primers described in [19]. All the polymerase chain reactions (PCRs) were carried out in a total volume of 25μL containing: 1μL DNA, 1x standard PCR buffer (Promega), 200μM of each dNTP, 1.5 mM MgCl_2_ (Promega), 0.1 μM of each primer and 1U of Taq DNA Polymerase (Promega). The amplicons were purified with ExoSAP-IT (GE Healthcare) and sequenced in both directions by using the ABI PRISM 3730 DNA Analyzer (Applied Biosystems, Foster City, California, USA) sequencer and the Sequential Big Dye Terminator 3.1 (Applied Biosystems, Foster City, California, USA). The program Geneious 7.1.3 was used to visualize the chromatograms.

For microsatellites, all 91 individuals were genotyped based on eight loci, previously described for *P*. *machrisii* species [20]. Amplification reactions were performed in 10μL, containing: 1μL of DNA, 1x standard buffer for PCR (Promega), 0.2mM of each dNTP, 0.25μM of each of the primers, 0.5U of Taq DNA polymerase (Promega) and 1.5mM of MgCl_2_ (Promega). The temperature profile employed was: 94 °C for 2 minutes; 35x (94 °C for 40 seconds, specific hybridization temperature for 40 seconds, 72 °C for 40 seconds); 72 °C for 10 minutes (S1 Table). Genotyping, was carried out through 6% PAGE (polyacrylamide gel electrophoresis), which is useful for separating small fragments of 1–500 base pairs (bp), making it possible to identify alleles. In order to avoid possible genotyping errors due to differences arising from the electrophoretic run and to facilitate the identification of alleles, individuals from all populations were included in the same gel for each locus. Genotypes were read on silver-stained-gels considering the diploid chromosome number in *P*. *aureispinus* [21]. For the IBO1 locality sample, the same microsatellite loci were previously genotyped through an automated sequencer [22], and alleles matching between the two methods were verified, ensuring the reproducibility of the genotypic data collected here.

### Data analysis of microsatellite markers

Micro-Checker 2.2.3 [23] was used to determine the possible genotyping errors due to the presence of null alleles and alleles drop-out or stutter bands. Linkage disequilibrium between loci was analysed with a likelihood ratio test implemented in ARLEQUIN 3.0 [24]. In order to avoid statistical type I error resulting from multiple testing, the sequential Bonferroni correction [25] was carried out independently for each population. Number of alleles per locus (*A*), effective alleles (*n*_E_), and private alleles, likewise observed (*H*_O_) and expected (*H*_E_) heterozygosities, inbreeding coefficient (*F*_IS_) and deviations from Hardy-Weinberg equilibrium (HWE) were estimated by using the software GENALEX 6.5 [26]. The sequential Bonferroni correction [25] was applied to the P-values of HWE tests, and to *F*_IS_ values per locus. In order to gain insights into the genetic diversity found for *P*. *aureispinus*, we used Wilcoxon’s rank sum tests to determine differences in genetic diversity between our estimates, those reported in microsatellite studies for threatened microendemic cactus species and for *campo rupestre* plant species. To this end, we surveyed the Web of Science database using the topic keywords ‘Cactaceae’ and ‘microsatellite’ to select studies investigating red-listed cactus species having similar geographic range to *P*. *aureispinus*, and the key words ‘microsatellite’ AND [‘campo*rupestre’ OR ‘rupestrian grassland’ OR "rock field’ OR "rocky savanna"] to select studies investigating plant species associated to the same habitats of *P*. *aureispinus*. Wilcoxon two-sample tests were performed on the by-locus (when available) or by-sample values of number of alleles per locus (*A*) and expected (*H*_E_) heterozygosity.

Level of population differentiation was assessed by using the *F*_ST_ fixation index, calculated on the estimator θ [27], and by the non-dependent within-population diversity statistics *G*”_ST_ [28] as implemented in GENELEX software 6.5 [26]. The software FREENA [29] was used to estimate *F*_ST_ values from corrected genotype frequencies to check possible overestimation of genetic differentiation induced by the presence of null alleles. We used ARLEQUIN 3.0 [24] to compute molecular variance (AMOVA) components among and within populations. We also explored the population genetic structure using different approaches. Bayesian analysis as implemented in STRUCTURE 2.3.4 [30] was used by assigning individual microsatellite genotypes to K gene clusters. An admixture model was run with correlated allele frequencies allowing the individuals to be assigned to two or more genotype groups when they are admixed. Each run was carried for 1,000,000 Markov Chain Monte Carlo (MCMC) cycles with a 10% burn-in. The algorithm was run 10 times for each value of K. The most likely number of clusters was inferred from ΔK statistics [31] as implemented in STRUCTURE HARVESTER 0.6.93 [32], by the smallest value of K after which the Ln P(D) values reach a plateau [30], and absence of ‘‘virtual” groupings, i.e. groups containing only individuals with genomes evenly scattered in more than one cluster. To further explore population structure in our microsatellite data, the Discriminant Analysis of Principal Components (DAPC) was performed in the R package adegenet [33]. After a preliminary run we retained 15 principal components (PC) that accounted for most of the total variance in the data (>85%), and a crescent number of clusters (K) from 2 to 15. We searched for the most likely number of groups through Bayesian Information Criterion (BIC) and the composition of individuals in each cluster. Script for DAPC analysis used is given in supporting information (S1 Information). Further, BARRIER 2.2 [34] was used to identify areas with genetic discontinuities among populations pairs using the *F*_ST_ matrix as genetic distance. The robustness of each inferred barrier was assessed by bootstrapping over loci using 100 simulated matrices of genetic differentiation. To test for evidences of population reduction, we used the heterozygosity-excess test implemented in BOTTLENECK 1.2.02 software [35].

The program GENECLASS2 [36] was used to detect migrants. First, the probability of an individual being a migrant from a non-sampled population was estimated. In a second step, the probability of an individual being a migrant from a sampled population was estimated by the ratio between the likelihood of an individual belonging to the population where it was sampled and the maximum likelihood value of that individual among the sampled populations. Both the analyses were performed by applying the Bayesian criterion defined by [37, 38] from 10,000 simulations with the alpha parameter set to 0.01. Isolation by distance (IBD) was tested using Mantel test [39] and canonical redundancy analysis (RDA), a method combining PCA and multiple regressions, which properly decompose the genetic variance based on allele frequencies [40]. For RDA, we used a script from [41] with slight modifications (the modified script is given as S2 Information) and the spatial component of the total genetic variation was obtained by multiplying the percentage of constrained variation by the overall value of *F*_ST_, as suggested [41].

### Assessment of conservation status

We assessed the criteria adopted by the International Union for Conservation of Nature [8] to identify the threat category from the known distribution of *P*. *aureispinus* (Fig 1) using two approaches. First, we used the online GeoCAT—Geospatial Conservation Assessment Tool (http://geocat.kew.org) software [42] to estimate the distribution area from the known occurrence points of the taxon based on two parameters: the extent of occurrence (EOO) and the area of occupancy (AOO). The extent of occurrence is calculated as the area of the minimum convex polygon that encompasses all the known occurrence points, and the area of occupancy is estimated by combining the areas of cells containing occurrence points (IUCN recommends 4km^2^ cells). We also estimated the potential EOO of *P*. *aurisetus* based on the association of this species with open habitats, especially those containing exposed rocks [11]. For this strategy, we downloaded Landsat 8 images (Fig 1B) from the USGS Global Visualization Viewer (available at: https://glovis.usgs.gov/next/) and calculated the Normalized Difference Vegetation Index (NDVI; [43] with ArcGIS 10.2 (Fig 1C and 1D). This index is calculated as the ratio from the Near Infrared and Red bands and varies from -1 (no vegetation) to 1 (healthy vegetation). Visual comparison of the natural colour image (Fig 1B) and NDVI (Fig 1C and 1D) allowed establishing thresholds for exposed soil and healthy vegetation. The NDVI layer was then used to define a threshold level that contained all known occurrence points and habitats with similar values for this index (potential EOO; Fig 1D).

## Results

### Plastid DNA markers

Although the plastid markers *trnT-trnL* and *psbD-trnT* were previously identified as highly variable in species of genus *Pilosocereus* ([15, 18] V.C. Dias, unpublished results) the sequencing results of plastid markers obtained in this study did not revealed any variation within and among localities. The obtained sequences of each plastid marker were submitted to GenBank (Accession Number: MF694640 to MF694645).

### Microsatellite loci

The program Micro-Checker recovered null alleles for all localities in *Pmac135* (21%) and *Pmac146* (31%) loci, which showed significant linkage disequilibrium after the Bonferroni sequential correction. Although the *F*_ST_ values for these two loci did not show significant difference when estimated from the original and corrected allele frequencies, as employed in FreeNA program, the inbreeding coefficient (*F*_IS_) in these loci was very different from others, causing a significant increase in mean *F*_IS_ values for the analysed samples. Because of these outlier results for the *Pmac135* and *Pmac146* we choose to remove these loci from the downstream analyses to avoid any important distortions in recovering the genetic diversity and population structure.

Based on the results of six loci (details of genotypes in S2 Table), the average number of alleles (*A*) ranged from 3.8 (IBO1) to 4.5 (IBO2); number of effective alleles (*n*_E_) ranged from 2.2 (IBO1) to 2.7 (OLB1). The observed mean heterozygosity (*H*_O_) ranged from 0.394 in IBO1 to 0.572 in OLB2, while the expected mean heterozygosity (*H*_E_) ranged from 0.390 in IBO1 to 0.525 in OLB1 (Table 1). Frequencies of private alleles range from 0.018 to 0.083 (S3 Table). Significant values of *F*_IS_ and significant deviations from HWE proportions after Bonferroni sequential correction were not found in any population.

**Table 1:** Genetic diversity parameters estimated for *P*. *aureispinus* on six microsatellite loci.

Locus	*N*	*A*	*n*_E_	*H*_O_	*H*_E_	*F*_IS_
	**Sample IBO1**					
*Pmac82*	24	2	1.1	0.083	0.080	-0.043
*Pmac84*	24	3	1.3	0.292	0.254	-0.147
*Pmac102*	24	1	1.0	0.000	0.000	N/D
*Pmac128*	24	5	3.3	0.667	0.702	0.051
*Pmac130*	24	8	4.2	0.625	0.760	0.178
*Pmac149*	23	4	2.2	0.696	0.542	-0.284
Overall loci (SE)	23.8 (0.1)	3.8 (1.0)	2.2 (0.5)	0.394 (0.13)	0.390 (0.13)	-0.049 (0.07)
	**Sample IBO2**					
*Pmac82*	27	3	1.1	0.074	0.072	-0.029
*Pmac84*	27	2	1.8	0.333	0.456	0.269
*Pmac102*	27	1	1.0	0.000	0.000	N/D
*Pmac128*	27	7	2.8	0.704	0.650	-0.082
*Pmac130*	27	9	5.6	0.889	0.821	-0.083
*Pmac149*	27	5	3.0	0.704	0.664	-0.060
Overall loci (SE)	27.0 (0.0)	4.5 (1.2)	2.5 (0.7)	0.451 (0.15)	0.444 (0.14)	0.003 (0.06)
	**Sample OLB1**					
*Pmac82*	28	3	1.5	0.429	0.357	-0.200
*Pmac84*	27	3	1.8	0.333	0.442	0.247
*Pmac102*	28	2	1.2	0.214	0.191	-0.120
*Pmac128*	28	6	4.2	0.750	0.760	0.013
*Pmac130*	28	8	5.4	0.964	0.816	-0.182
*Pmac149*	27	4	2.4	0.741	0.585	-0.266
Overall loci (SE)	27.6 (0.2)	4.3 (0.9)	2.7 (0.7)	0.572 (0.12)	0.525 (0.10)	-0.085 (0.07)
	**Sample OLB2**					
*Pmac82*	12	2	1.2	0.167	0.153	-0,091
*Pmac84*	12	4	1.8	0.583	0.451	-0,292
*Pmac102*	12	3	1.4	0.167	0.292	0,429
*Pmac128*	12	6	3.9	0.833	0.747	-0,116
*Pmac130*	11	7	4.7	1.000	0.789	-0,267
*Pmac149*	11	4	2.1	0.364	0.533	0,318
Overall loci (SE)	11.6 (0.2)	4.3 (0.7)	2.5 (0.6)	0.519 (0.14)	0.494 (0.10)	-0.003 (0.12)

*N* indicates sample size; *A*, number of alleles per locus; *n*_E_, effective number of alleles; *H*_O_, observed heterozygosity; *H*_E_, expected heterozygosity; *F*_IS_, inbreeding coefficient; N/D, non-determined; SE, standard error.

Ten studies investigating eight cactus species red-listed by IUCN and eight studies investigating 11 plant species from *campos rupestres* were selected from our surveys on the Web of Science (2017 Dec 4, data not shown) for genetic diversity comparisons (Table 2). Most of the red-listed cactus species are microendemics from central Mexico, and two species also occur in the *campos rupestres* from eastern Brazil. Overall, we found high genetic diversity reported for those species, with mean values of *H*_E_ and *A* ranging from 0.465 and 3.8 in *P*. *parvus* [22] to 0.765 and 8.8 in *Mammillaria crucigera* [44]. Among the selected *campo rupestre* plants, there are six cactus species, of which five belong to the genus *Pilosocereus*. The remaining plants include one species of each family Euriocaulaceae, Melastomataceae, Orchidaceae, Polygonaceae, and Velloziaceae. Genetic diversity in this group was moderate to high, with mean values of *H*_E_ and *A* ranging from 0.324 to 0.753 and from 2.7 to 9.7, respectively. The Wilcoxon tests indicated that the number of alleles and the expected heterozygosity in *P*. *aureispinus* were significantly smaller than those reported for five of the eight surveyed cactus species, including the *campo rupestre* species *Uebelmannia pectinifera*. Comparing with other *campo rupestre* plant species, genetic diversity estimates for *P*. *aureipsinus* were not significantly different from eight of the 11 surveyed species, including five *Pilosocereus* species, being only smaller than those reported for two species of the families Eriocaulaceae and Velloziaceae.

**Table 2:** Mean allele diversity and expected heterozygosity reported for both microendemic threatened cactus species^1^ and for *campo rupestre* plant species^2^ in studies using microsatellite markers till December 2017*. The results of Wilcoxon’s rank sum tests (WRST) comparing whether the genetic diversity estimates of *Pilosocereus aureispinus* is significantly (*P* ≤ 0.05) larger (>), smaller (<) or not different (≈) to the reported species is shown.

Family/Species	*A* (SE)^#^	WRST(*A*)	*H*_E_ (SE)^#^	WRST(*H*_E_)	Study
**Cactaceae**					
^1^*Astrophytum asterias*	5.9 (1.6)	<	0.695 (0.069)	<	Terry et al. 2012 [63]
^1^*Astrophytumasterias*	8.5 (3.4)	<	0.632 (0.180)	≈	Terry et al. 2006 [64]
^1^*Ariocarpusbravoanus*	5.6 (1.2)	≈	0.511 (0.160)	≈	Hugues et al. 2008 [65]
^1^*Echinocactus grusonii*	3.3 (1.6)	≈	0.508 (0.160)	≈	Hardesty et al. 2008 [66]
^1^*Mammillaria crucigera*	8.8 (3.4)	<	0.758 (0.158)	<	Solórzano et al. 2009 [44]
^1^*Mammillaria crucigera*	8.0 (2.1)	<	0.765 (0.060)	<	Solórzano and Dávila 2015 [67]
^1^*Mammillariapectinifera*	n/a	n/a	0.772 (0.022)	<	Maya-García et al. 2017 [68]
^1^*Mammillariasupertexta*	8.2 (0.9)	<	0.764 (0.024)	<	Solórzano et al. 2014 [69]
^1,2^*Pilosocereusaureispinus*	4.0 (2.3)	n/a	0.463 (0.276)	n/a	This work
^2^*Pilosocereusaurisetus*	5.4 (3.4)	≈	0.551 (0.293)	≈	Bonatelli et al. 2014 [15]
^2^*Pilosocereusmachrisii*	4.4 (3.1)	≈	0.478 (0.288)	≈	Bonatelli et al. 2014 [15]
^2^*Pilosocereusjauruensis*	4.2 (2.6)	≈	0.488 (0.270)	≈	Bonatelli et al. 2014 [15]
^1,2^*Pilosocereusparvus*	3.8 (1.3)	≈	0.465 (0.230)	≈	Moraes et al. 2012 [22]
^2^*Pilosocereusvilaboensis*	4.2 (3.2)	≈	0.438 (0.320)	≈	Bonatelli et al. 2014 [15]
^1,2^*Uebelmanniapectinifera*	6.0 (2.5)	<	0.690 (0.135)	<	Moraes et al.2014 [70]
**Eriocaulaceae**					
^2^*Comanthera elegans*	7.9 (4.0)	<	0.663 (0.210)	<	Leal et al. 2014 [71]
**Melastomataceae**					
^2^*Tibouchina papyrus*	n/a	n/a	0.324 (0.106)	≈	Collevatti et al. 2012 [72]
**Orchidaceae**					
^2^ *Cattleyabrevipedunculata*	9.7 (1.5)	≈	0.720 (0,051)	≈	Leal et al. 2016 [73]
**Polygonaceae**					
^2^*Coccolobacereifera*	2.7 (0.3)	>	0.460 (0.076)	≈	Moreira et al. 2010 [74]
**Velloziaceae**					
^2^*Velloziagigantea*	6.1 (1.5)	<	0.753 (0.180)	<	Martins et al. 2012 [75]

*, two studies in this category [76, 77], dealing with the triploid species *Haageocereus tenuis* Ritter were not included here;

^#^, mean and SE were calculated from by-locus (when available) or by-sample values reported in each study; *A*, mean number of allele per locus; *H*_E_, expected heterozygosity; SE, standard error; n/a, not available or not applicable.

The overall *F*_ST_ for all the four localities obtained by the θ estimator [27] was 0.071, but ranged from 0.055 (*Pmac130*) to 0.095 (*Pmac149*) per locus. Similarly, the *G*"_ST_ index resulted in an average value of 0.172 ranged from 0.061 (*Pmac82*) to 0.295 (*Pmac128*) (Table 3).

**Table 3:** Population differentiation estimates per loco.

Locus	*F*_ST_	*P*-value	*G’*’_ST_	*P*-value
*Pmac82*	0.055	0.007	0.061	0.009
*Pmac84*	0.069	0.019	0.112	0.018
*Pmac102*	0.068	0.005	0.068	0.005
*Pmac128*	0.078	0.002	0.295	0.002
*Pmac130*	0.051	0.001	0.237	0.001
*Pmac149*	0.095	0.001	0.248	0.001
Overall	0.070	0.005	0.172	0.006

According to STRUCTURE approach, a single genetic cluster (K = 1) is the most likely hypothesis to explain the distribution of genetic variation in our samples. The validity of the K = 1 hypothesis was evaluated through the Ln P(D) values of the different K values tested (S2 Fig) and by the presence of ‘‘virtual” groupings for K = 2. This hypothesis was further supported by the DAPC analysis, in which no geographic pattern emerged. The lower BIC values in the DAPC analysis corresponded to five to 11 clusters with all clusters grouping individuals from different sites. Even for K = 2, which showed a clear cut among clusters, individuals from all (except OLB2) sites were assigned to different clusters by DAPC, indicating no geographic structure in the data (S3 Information). A hierarchical AMOVA grouping populations in two geographic groups (IBO1-IBO2 and OLB1-OLB2) also supported the one genetic group hypothesis, as the variance component between the two population groups was statically zero (Table 4). In contrast with these results, BARRIER inferred the existence of three well-supported spatial barriers (bootstrap values = 100%; S3 Fig) to gene flow isolating each sampling site.

**Table 4:** Results of AMOVA on the microsatellite variation of *P*. *aureispinus*.

Hypothesis	Source of Variation	% variation	*F*-statistic	*P*-value
Single population	Among sites	3.07	*F*_ST_ = 0.03	0.000
	Within sites	96.93		
Two groups (IBO1-IBO2; OLB1-OLB2)	Among groups	-0.94	*F*_CT_ = -0.009	0.000
	Among sites within groups	7.50	*F*_SC_ = 0.07	0.000
	Within sites	93.45	*F*_ST_ = 0.06	0.000

The RDA analysis and Mantel test (r2 = -0.1699; P = 0.29, S1 Fig) did not support any spatial correlation between the genetic and geographic distances among populations. GENECLASS2 program showed four possible migrant individuals in the IBO1 population (S21A17) from the OLB1 population, a possible IBO2 individual (S49A21) from IBO1, and two individuals in the population OLB2 (S118A1 and S119A2) originating from IBO2 (Table 5). No significant (P ≤ 0.01) excess of heterozygotes were detected with BOTTLENECK for all populations (P_(IBO1)_ = 1.00, P_(IBO2)_ = 0.81, P_(OLB1)_ = 0.04, P_(OLB2)_ = 0.56; Wilcoxon test), indicating that the populations did not undergo a recent bottleneck.

**Table 5:** Results of the migrant detection analysis.

Migrant Individual	Sampling Population	GENECLASS*
S21A17	IBO1	OLB1
S21A20	IBO1	OLB1
S49A1	IBO2	IBO1
S49A2	IBO2	OLB1
S118A1	OLB2	IBO2
S118A2	OLB2	unknown

*The probable source populations according to GENECLASS [36] estimates.

### Conservation status

The results obtained in GeoCAT assessed the species as endangered (EN). According to this analysis, the species shows extremely limited EOO (app. 300 Km^2^) and AOO (20 Km^2^ using 4km^2^ grid cells). Our strategy to estimate the potential EOO from the NDVI layer resulted in a larger area (app. 750 Km^2^) than obtained with GeoCAT. However, both estimates are within the thresholds for Endangered species (EOO < 5,000 km^2^ and AOO < 500 km^2^; IUCN 2017). Moreover, the species has been found in just five different localities, in which two of them (OLB1 and OLB2) are only 5 km apart.

## Discussion

Our expectations of the low levels of genetic diversity and some degree of population structure in the threatened and microendemic species *P*. *aureispinus* were not completely met in this study. The investigated populations showed moderate levels of genetic diversity, with mean values of *H*_E_ and *A* ranging from 0.324 to 0.753 and from 2.7 to 9.7, respectively. These estimates of genetic diversity were generally smaller than those reported for other microendemic and threatened cactus species (Table 2). The genetic diversity in *P*. *aureispinus* was statistically similar with only two microendemic cactus species, both from Mexican dry lands (*Ariocarpus bravoanus* and *Echinocactus grusonii*) and with the congeneric *P*. *parvus* growing in *campos rupestres* of eastern Brazil. This picture changes when comparisons are made with both cactus and non-cactus species restricted to *campo rupestre* landscapes. Our results showed that the genetic diversity in *P*. *aureispinus* was lower in comparison with only two *campo rupestre* species, *Comanthera elegans* and *Vellozia gigantea*. In contrast, our genetic diversity estimates were comparable to three other non-cactus species and to five cactus species from genus *Pilosocereus*, including species as *P*. *machrisii* and *P*. *aurisetus* and the Melastomataceae *Tibouchina papyrus* with wide-range distributions. Taken together, these results suggest that *P*. *aureispinus* is not experiencing genetic erosion due to its restricted range; the level of genetic diversity is instead likely related to common characteristics affecting the genetic diversity both in *Pilosocereus* and *campo rupestre* species. For instance, *campo rupestre* landscapes has been recently proposed as an old climatically-buffered infertile landscape (OCBIL; [12]). The OCBIL theory predicts, among other things, effective cross-pollination by highly mobile vectors as a key process favouring genetic variation and connectivity among spatially-disjunct populations of OCBIL endemic species [45]. However, the gene dispersal vectors are unknown for most surveyed species in Table 2, preventing observations that could support this OCBIL prediction for *campo rupestre* landscapes.

In contrast to microsatellite variation found in *P*. *aureispinus*, plastid markers (*trnT-trnL* and *psbD-trnT*) did not show any variation. Besides recent selective sweep, a possible explanation to the lack of variation on the plastid markers could be a recent bottleneck of *P*. *aureispinus*. However, the moderate variation in nuclear markers and the absence of significant excess of heterozygotes prevent this later possibility. An alternative explanation could be a long-term effect of genetic drift eroding variation mostly in plastid genome, as cytoplasmic genomes in general presents fourfold smaller effective population size than nuclear genomes. Although previous studies have found intraspecific variation on cactus species in the plastid markers used in this study [46], including *Pilosocereus* species [18], lineage-specific variation in cpDNA has been reported in Cactaceae, even among closely related species [47]. These findings suggest that other plastid regions of *P*. *aureispinus* could exhibit some level of variation.

*Pilosocereus aureispinus* only occurs in patches of rocky outcrops in a rugged topographic region, which may restrict connectivity in this species. Even with this patchy distribution, all analyses (except BARRIER) found no clear population genetic structure in our data. The results obtained after excluding the potentially biased loci (*Pmac135* and *Pmac146* loci) indicated low genetic differentiation among populations, suggesting high level of recent gene flow according to the lower *F*_ST_ and *G*"_ST_ and small number of low-frequency private alleles. Further, STRUCTURE, DAPC, and AMOVA results suggested that *P*. *aureispinus* is composed of a single population group. In contrast, BARRIER was able to infer barriers to gene flow isolating each sampling site. Because overall and pairwise *F*_ST_ were low and taking into account the previous results of our population structure analyses, we conclude that BARRIER has identified a subtle, more sensible restriction to gene flow among populations. Further the analysis implemented in GENECLASS2 detected four possible migrants in 91 individuals (Table 3), indicating that seed and/or pollen movement among all the localities may eventually occur. Although very little is known about the reproductive biology of *P*. *aureispinus*, its floral characteristics such as nocturnal anthesis, short perianth-segments, and robust flowers resistant to the impact caused by visitors, suggest bats as its primary pollinator. Bat-mediated gene dispersal has been associated as an important factor contributing to low population structure and high genetic diversity in other cactus species (e.g. [48] and references therein). Although cactus species generally have flowers phenotypically specialized to certain pollination types [49], the systems closely studied so far actually work as generalists [50]. Contrary to the species floral guilds of pollination, if *P*. *aureispinus* is generalist then the K = 1 structuring is likely maintained by efficient seed and/or pollen dispersal vectors. Besides the effectiveness of seed and/or pollen movement connecting the cactus patches, an alternative (and no exclusive) explanation to the lack of marked genetic structuring in *P*. *aureispinus* are the past events of expansion during glacial times that *Pilosocereus* species have experienced [15], which could have increased the connectivity among the cactus patches.

In plants, the mating system, pollination syndrome, seed dispersal, longevity of individuals and clonal reproduction are important factors influencing the level, maintenance, and distribution of genetic variation within and between populations [51]. For instance, long-lived species generally exhibit higher levels of genetic variation compared to short-lived species because of the longevity of genotypes [52]. Further, the maintenance of different genotypes through many generations by long-term persistence of plants exhibiting vegetative reproduction can increase effective population sizes by decreasing the rate at which alleles are lost, ultimately increasing the heterozygosity [53, 54]. As observed in Cactaceae, *P*. *aureispinus* plants can reproduce vegetatively when detached cladodes become rooted in the ground. Thus, besides different possible explanations towards the high genetic diversity and lack of genetic structuring in *P*. *aureispinus* i.e., efficient seed and pollen dispersal vectors, clonality and longevity must represent important factors in the maintenance of genetic diversity in this species.

The results from the EOO analyses were not concordant with the current classification of the species as vulnerable (VU). According to the current known distribution both methods used here suggested that the species should be raised to the Endangered (EN) category. This change of status is in consonance with the prominent phylogenetic distinctiveness of *P*. *aureispinus* as an early divergent lineage within the *Pilosocereus* subgenus [15, 16]. A visual comparison of the results obtained to estimate the EOO of *P*. *aureispinus* from GeoCAT and using the NDVI layer, indicates that the latter was probably more accurate to propose suitable areas for the species occurrence (Fig 1). This larger potential distribution indicates that the species is present in other sites than the five occurrence sites known until now and it could be used as a field-guide to find more natural populations of *P*. *aureispinus* within this area. The use of NDVI to assess species distribution has been suggested by several authors [55, 56 and references therein], and showed high predictive results in *Coccoloba cereifera* [57], a species that also grows in *campo rupestre*. Indeed, because the high colour contrast between rocky and forested landscape in satellite images, the use of NDVI to estimate EOO in *campo rupestre* species seems to be a compelling approach.

Species distribution ranges are usually addressed as one of the main factors to explain the level of genetic variability found in natural populations. Species with a wide and continuous distribution are supposed to have higher genetic diversity than those with restricted or endemic distribution [58]. However, the general assumption that narrow endemic species encompass low genetic diversity [4] is not always observed in plants, as rare species have already shown to have higher genetic diversity than their congeneric widespread ones [59, 60, 61]. Further, this and other studies with different cactus species have suggested an opposite pattern than previously expected (Table 2).

## Conclusions

To the best of our knowledge, this is the first population genetic study on a species growing in arenitic rock outcrops from Brazilian Cerrado/Caatinga boarder. In contrast with our expectation of low genetic diversity in *P*. *aureispinus*, the genetic diversity found for this species is comparable with widespread and closely allied species [22], at least considering the microsatellite DNA. Further, even occupying a rugged topographic region, which may restrict connectivity, the results obtained here suggest that the currently known localities of *P*. *aureispinus* present low genetic structure. The moderate genetic diversity found in *P*. *aureispinus* indicates that pollination and/or seed dispersal are efficient and promote gene flow between the localities, increasing the general effective size and reducing the impact of genetic drift. Further we concluded that this species is not suffering from deleterious genetic effects expected for species with restricted distribution, at least on the nuclear genome. Although the species has moderate to high genetic diversity, considering the extremely narrow inferred and known distribution range and its phylogenetic distinctiveness [62] we advocate that *P*. *aureispinus* should be considered an endangered species. In order to protect against loss of genetic variability and extinction of this species, seeds are needed to be collected from as many individuals as possible and stored in seed banks. Moreover, *P*. *aureispinus* needs to be federally listed as an endangered species.

## Supporting information

S1 Table Details of microsatellite loci used for genotyping in *P*. *aureispinus*. (DOCX)

S2 Table Genotyping information of 91 individuals for *P*. *aureispinus* based on six microsatellite loci. (XLSX)

S3 Table List of private alleles by population/locus and their respective frequencies. (DOCX)

S1 Fig Mantel test RMA regression results. (DOCX)

S2 Fig Ln P(K) distribution using the “log probability of data” (Mean of LnP±1) approach estimated by means of 10 replications in STRUCTURE. (DOCX)

S3 Fig Map showing the results of BARRIER analysis. (DOCX)

S1 Information Script for Discriminant Analysis of Principal components (DAPC). (R)

S2 Information Script for redundancy analysis (RDA). (R)

S3 Information Results from DAPC analysis retaining the first 15 PCs. (DOCX)
