# Supplementary material for: Weak population structure and no genetic erosion in Pilosocereus aureispinus: A microendemic and threatened cactus species from eastern Brazil
Source: PLoS One. 2018 Apr 9;13(4):e0195475. doi: 10.1371/journal.pone.0195475 (PMC5890996; doi:10.1371/journal.pone.0195475)
Supplement: S1 Table — (DOCX) [file pone.0195475.s001.docx]

| **S1 Table** Details of microsatellite DNA loci used for genotyping in *P. aureispinus*, their repetitive motifs, sequences of forward (F) and reverse (R) primer, and annealing temperatures (Ta). | | | | |
| --- | --- | --- | --- | --- |
| Loci | Repetitive Motifs | Sequences | | Ta(^o^ C) |
| *Pmac082* | (GAG)_5_(GAA)_2_  (GAG)_2_GAA(GAG)_2_ | | F: GTAAAAGAGGAGGATGGAGAGG  R: CTTCTTCTTGCTAGGTTTCTCG | 56 |
| *Pmac084* | (AG)_9_CG(AG)_2_ | | F: CATAAATTGCAGAAATGAGGAC  R: AGGTAAACCGCTAACTCGATG | 52 |
| *Pmac102* | (AG)_9_ | | F: TCTATAAGTGCCGATGGATGC  R: CACACCTCACTCCCAACCTC | 59 |
| *Pmac128* | (TC)_5_TT(TC)_10_ (AC)_10_ | | F: GTGTTGATTGTACTCTTCAG  R: CTAACCCTTTGTATACATGC | 58 |
| *Pmac130* | (AG)_7_CA(AG)_12_ | | F: GAGGTGCCAATAAATCG  R: TGTCACGCAATCTTGAACC | 55 |
| *Pmac135* | (TC)_5_TG(TC)_12_ | | F:ACCAGAATGAGCTCAGCTGTAG R:CCTAGCTAGCAGAATCAGAGTGAAGAC | 54 |
| *Pmac146* | (AG)_20_ | | F: ACCCGACATCCCACTTGTAG  R: TAGTCTGAAACGGAGCAAGG | 60 |
| *Pmac149* | (TC)_19_ | | F: TTCATCCTGCTTTTGAAGTTTG  R: TGATGGATTAGGATTGACCTG | 59 |
