# Supplementary material for: Weak population structure and no genetic erosion in Pilosocereus aureispinus: A microendemic and threatened cactus species from eastern Brazil
Source: PLoS One. 2018 Apr 9;13(4):e0195475. doi: 10.1371/journal.pone.0195475 (PMC5890996; doi:10.1371/journal.pone.0195475)
Supplement: S3 Table — (DOCX) [file pone.0195475.s003.docx]

| **Table S3** List of private alleles by population/ locus and their respective frequencies | | |
| --- | --- | --- |
| Population (Locus) | Alelle | Frequency |
| IBO1 (*Pmac128*) | 122 | 0.042 |
| IBO2 (*Pmac128*) | 120 | 0.037 |
| OLB1 (*Pmac82*) | 96 | 0.071 |
| OLB1 (*Pmac128*) | 134 | 0.018 |
| OLB2 (*Pmac102*) | 104 | 0.083 |
| OLB2 (*Pmac128*) | 124 | 0.083 |
