# Supplementary material for: Weak population structure and no genetic erosion in Pilosocereus aureispinus: A microendemic and threatened cactus species from eastern Brazil
Source: PLoS One. 2018 Apr 9;13(4):e0195475. doi: 10.1371/journal.pone.0195475 (PMC5890996; doi:10.1371/journal.pone.0195475)
Supplement: S1 Fig — (DOCX) [file pone.0195475.s004.docx]

**
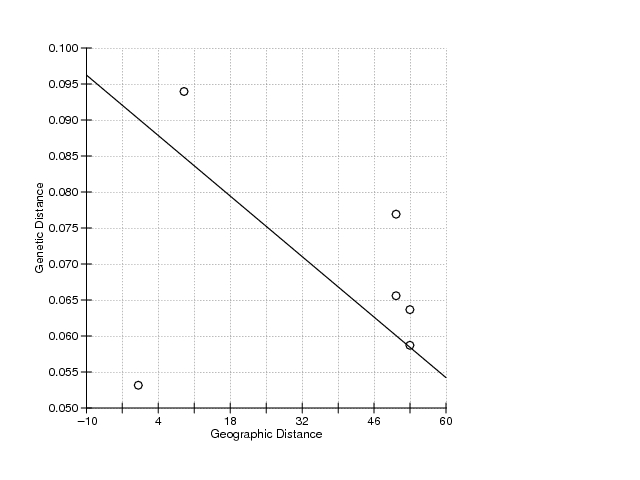
**

**S1 Fig.** Mantel test RMA regression line between genetic distance (F_ST_) and geographical distance (km) among 4 populations of *Pilosocereus aureispinus* based on “Isolation by Distance” analyses (r2 = -0.1699; P = 0.29)
