# Supplementary material for: Weak population structure and no genetic erosion in Pilosocereus aureispinus: A microendemic and threatened cactus species from eastern Brazil
Source: PLoS One. 2018 Apr 9;13(4):e0195475. doi: 10.1371/journal.pone.0195475 (PMC5890996; doi:10.1371/journal.pone.0195475)
Supplement: S2 Fig — (DOCX) [file pone.0195475.s005.docx]

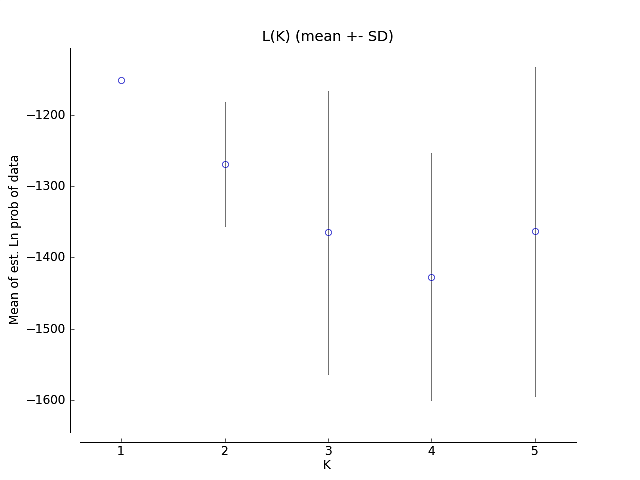


**S5 Fig**: Ln P(K) distribution using the “log probability of data” (Mean of LnP±1) approach estimated by means of 10 replications in STRUCTURE.
