# Supplementary material for: Weak population structure and no genetic erosion in Pilosocereus aureispinus: A microendemic and threatened cactus species from eastern Brazil
Source: PLoS One. 2018 Apr 9;13(4):e0195475. doi: 10.1371/journal.pone.0195475 (PMC5890996; doi:10.1371/journal.pone.0195475)
Supplement: S3 Fig — (DOCX) [file pone.0195475.s006.docx]

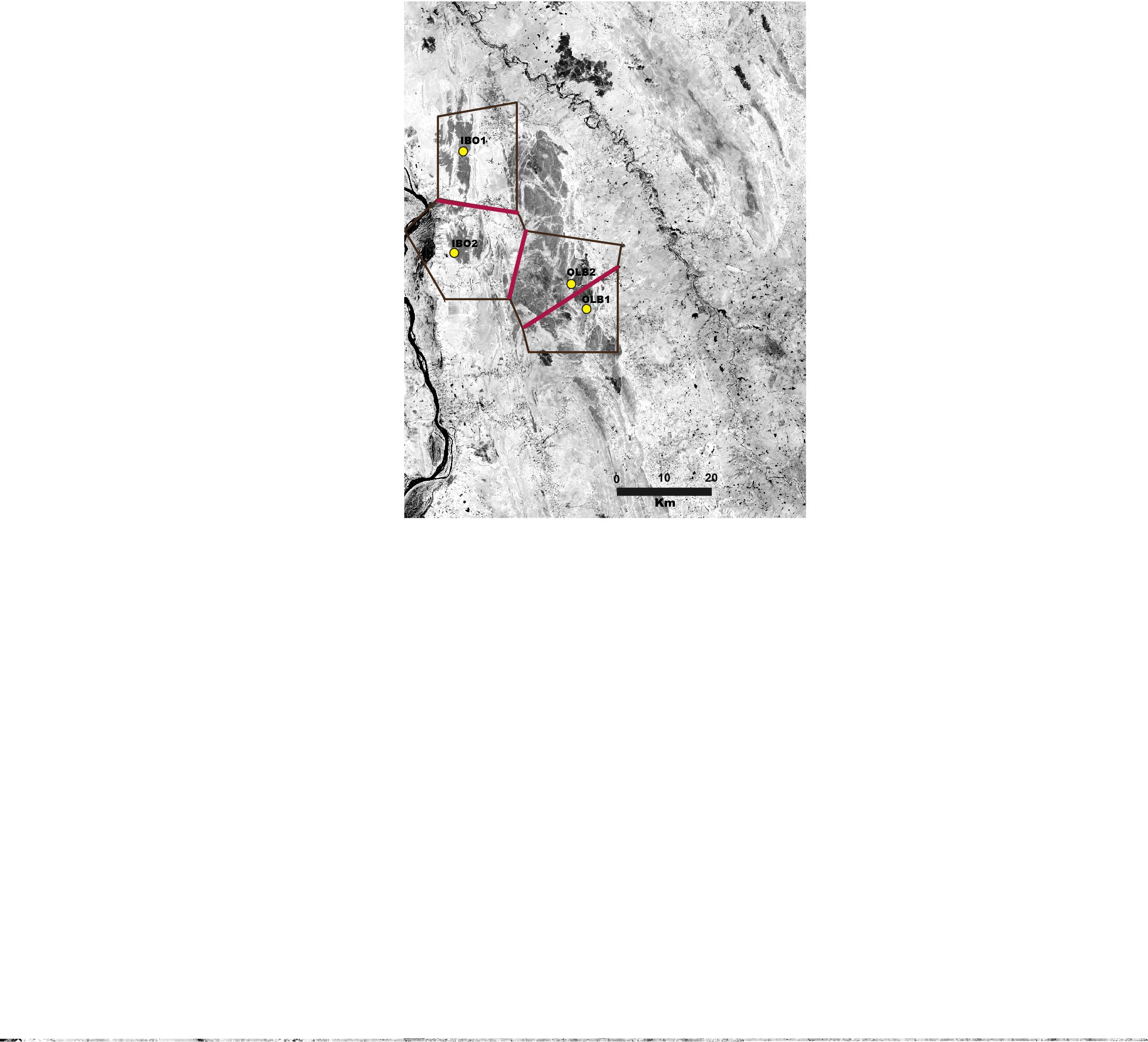


**S3 Figure**. Spatial genetic discontinues identified by the Monmonier’s algorithm implemented in Barrier 2.2. Polygons are the result of the Voronoi tessellation for each population and red lines represent supported spatial barriers (bootstrap = 100%) between populations.
