# Supplementary material for: Weak population structure and no genetic erosion in Pilosocereus aureispinus: A microendemic and threatened cactus species from eastern Brazil
Source: PLoS One. 2018 Apr 9;13(4):e0195475. doi: 10.1371/journal.pone.0195475 (PMC5890996; doi:10.1371/journal.pone.0195475)
Supplement: S3 Information — (DOCX) [file pone.0195475.s009.docx]

**S3 Information**. Results from DAPC analysis retaining the first 15 PCs.


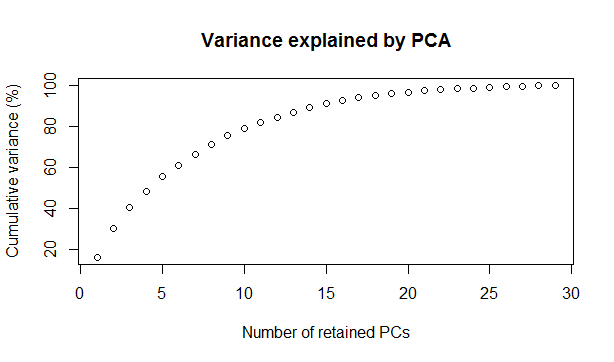


Figure 1: Cumulated variance explained by the eigenvalues of the PCA in DAPC analysis.


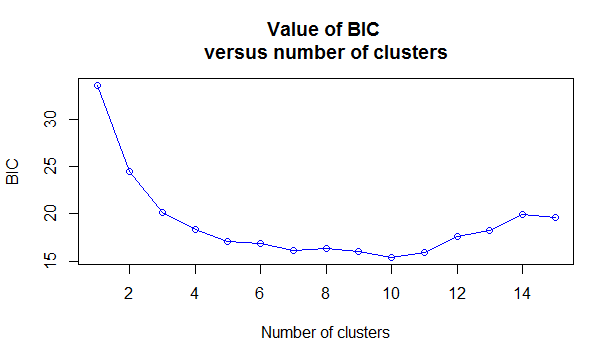


Figure 2: Bayesian Information Criterion (BIC) values for different values of k using the first 15 PCs in DAPC analysis.

**
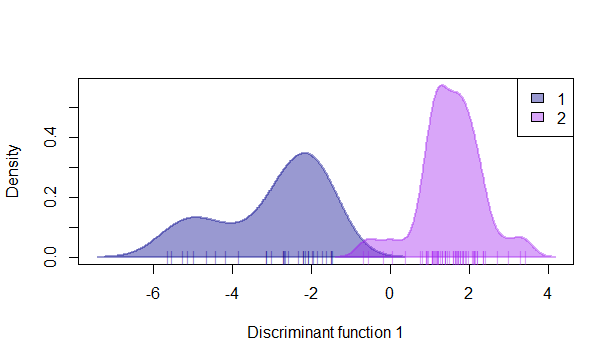
**

Figure 3: Distribution of membership probabilities (density) of individuals for K = 2 based on the first discriminant function of DAPC analysis.

Table 4: Group composition of individuals assigned by DAPC for different K =2

|  |  | K2 |  |  |  |
| --- | --- | --- | --- | --- | --- |
| Code of Population | Group 1 | Group 2 |  |  |  |
| IBO 1 | 19 | 5 |  |  |  |
| IBO2 | 13 | 14 |  |  |  |
| OLB 1 | 16 | 12 |  |  |  |
| OLB2 | 12 | 0 |  |  |  |
